# Toward Universal Protection: A Comprehensive Review of Pneumococcal Disease, Emerging Vaccination Challenges and Future Perspectives

**DOI:** 10.3390/vaccines13121237

**Published:** 2025-12-12

**Authors:** Mayla Sgrulletti, Maria Felicia Mastrototaro, Alessandra Beni, Gloria Mantuano, Giorgio Costagliola, Veronica Santilli, Davide Montin, Caterina Rizzo, Baldassarre Martire, Gian Luigi Marseglia, Michele Miraglia Del Giudice, Viviana Moschese, Immunology (SIAIP) Vaccine Committee

**Affiliations:** 1 Pediatric Immunopathology and Allergology Unit, Policlinico Tor Vergata, University of Rome Tor Vergata, 00133 Rome, Italy; 2 Pediatrics and Neonatology Unit, Maternal-Infant Department, “Monsignor A.R. Dimiccoli” Hospital, 70051 Barletta, Italy; 3 Department of Clinical and Experimental Medicine, University of Pisa, 56126 Pisa, Italy; 4 Department of Pediatrics, University of Bari “Aldo Moro”, 70122 Bari, Italy; 5 Section of Pediatric Hematologyand Oncology, Azienda Ospedaliero Universitaria Pisana, 56126 Pisa, Italy; 6 Research Unit of Clinical Immunology and Vaccinology, Academic Department of Pediatrics (DPUO), IRCCS Bambino Gesù Children’s Hospital, 00165 Rome, Italy; 7 Division of Pediatric Immunology and Rheumatology, “Regina Margherita” Children Hospital, 10126 Turin, Italy; 8 Department of Translational Research and New Technologies in Medicine and Surgery, University of Pisa, 56126 Pisa, Italy; 9 Pediatric Unit, Department of Clinical, Surgical, Diagnostic, and Pediatric Sciences, University of Pavia, 27100 Pavia, Italy; 10 Pediatric Clinic, Fondazione IRCCS Policlinico San Matteo, 27100 Pavia, Italy; 11 Department of Woman, Child and General and Specialized Surgery, University of Campania “Luigi Vanvitelli”, 81100 Naples, Italy

**Keywords:** pneumococcal diseases, streptococcus pneumoniae, pneumococcal conjugate vaccines, serotype replacement, diagnostic vaccination, inborn errors of immunity, primary antibody deficiency, specific antibody deficiency, novel vaccine technology, universal vaccine

## Abstract

*Streptococcus pneumoniae* contributes significantly to morbidity, mortality, and healthcare costs worldwide due to severe Invasive Pneumococcal Disease (IPD), particularly among young children and vulnerable populations. This review critically examines the current state of pneumococcal disease epidemiology, the evolution of vaccine strategies, and persistent challenges to achieve global control of the disease. The implementation of Pneumococcal Conjugate Vaccines (PCVs) has yielded substantial public health gains, establishing herd protection and sharply reducing vaccine-type IPD incidence. However, this success has been fundamentally challenged by serotype replacement, where non-vaccine serotypes have subsequently emerged to cause a significant proportion of the residual disease burden. This epidemiological shift has necessitated the development and deployment of higher-valency PCVs (PCV15, PCV20, and PCV21) to expand serotype coverage. Furthermore, optimal protection requires personalized strategies for high-risk cohorts where vaccine effectiveness can be compromised. In this context, the review details how pneumococcal vaccination—and particularly PPSV23—serves as an indispensable diagnostic tool to evaluate a broad spectrum of Inborn Errors of Immunity (IEI) and in particular humoral defects. Diagnostic challenges are strained by non-standardized assays and the limited panel of unique serotypes available for testing in the PCV era. The scientific priority is now the development of universal protein-based vaccines, to provide protection against all serotypes and non-encapsulated strains by targeting conserved virulence factors. This integrated approach, combining expanded PCV coverage with novel vaccine technology, is essential to mitigate the ongoing public health burden of pneumococcal disease.

## 1. Burden of Pneumococcal Disease in Children

*Streptococcus pneumoniae*, also known as pneumococcus, was first isolated by Louis Pasteur in 1881. It is a Gram-positive, α-hemolytic, facultative anaerobic bacterium that is specific to humans. Most strains are encapsulated by polysaccharides, with these capsular polysaccharides (CPs) serving as major virulence factors and the basis for serotype classification [1]. Pneumococcal disease is endemic globally, with over 100 identified serotypes, many of which are targeted by current vaccines. However, only a subset of these serotypes is responsible for the majority of pneumococcal infections worldwide.

The clinical impact of *Streptococcus pneumoniae* varies by serotype. Some serotypes are more likely to cause severe disease, while others are primarily associated with asymptomatic colonization of the nasopharynx. Asymptomatic carriage is especially common in children and varies significantly by region, with prevalence rates ranging from 3% to 50% in healthy preschool-aged children, and 5–10% in healthy adults. Approximately 20 to 25 predominant serotypes are responsible for nearly 90% of invasive pneumococcal disease cases [1]. *Streptococcus pneumoniae* is one of the leading bacterial causes of community-acquired pneumonia (CAP) in both children and adults. It is capable of causing a wide spectrum of diseases, ranging from serious invasive pneumococcal diseases (IPD)—such as meningitis and bacteremic pneumonia—to less severe, non-invasive infections like acute otitis media (AOM), sinusitis, and non-bacteremic pneumonia (non-IPD) [2].

The prevalence and distribution of the most virulent serotypes vary by age, geographical location, clinical manifestation, and antibiotic use [3].

A review by Yadong et al. identified serotype 19A as one of the most commonly found across all geographic regions. Additionally, serotypes 1 and 14 were prominent in Europe and Latin America, while serotypes 6B, 14, and 19F were significant in Africa and the Eastern Mediterranean [4]. A 2014 systematic review focusing on South Asian Association for Regional Cooperation (SAARC) countries found distinct dominant serotypes in children under 12 years: serotype 1 in Nepal, serotype 14 in Bangladesh and India, and serotype 19F in Sri Lanka and Pakistan [5].

In Europe in 2022, for IPD cases in infants under one year of age, serotypes 3, 19A, 8, 10A, and 24F were predominant. For the one- to four-year age group, serotypes 19A and 3 were the most common [6].

### Epidemiology of Different Serotypes in Both High-Income and Low-and Middle-Income Countries

Pneumococcal disease (PD) continues to pose a significant global health challenge, disproportionately affecting non-high-income countries (NHICs) where it accounts for a substantial portion of the global burden and most PD-related deaths. It is one of the leading contributors to vaccine-preventable morbidity and mortality worldwide, with the highest burden found in young children (aged ≤ 5 years), older adults (aged ≥ 65 years), and individuals with underlying medical conditions. The World Health Organization (WHO) estimates that PD is responsible for over 300,000 deaths annually in children under five years of age worldwide [7]. Globally, over 1.2 million deaths occurred in children under five in 2015, with 25% attributed to IPD. In 2015, the Southeast Asian region recorded the highest incidence of PD in children under five, at 2509 cases per 100,000 children. India, for instance, reported an IPD incidence of 17.8 per 100,000 in children under five, with a peak of 49.9 per 100,000 in infants aged 6–12 months. Similarly, in Pakistan, IPD was estimated to cause 22% of all mortality in children under five [8]. In Europe, the crude IPD notification rate in 2022 was 5.1 cases per 100,000 population, peaking at 13.4 cases per 100,000 in infants under the age of one. Notification rates varied considerably across countries, from 0.1 to 12.2 cases per 100,000, reflecting both genuine differences in disease prevalence and inconsistencies in surveillance strategies [6]. While IPD presents a severe threat, the incidence of non-invasive pneumococcal disease is even higher. For example, in the United States (US), 60% of children experience at least one episode of AOM by age three and AOM is the leading cause of pediatric outpatient visits and antibiotic prescriptions in the US [9].

PD is associated with substantial direct medical costs, largely due to the frequent hospitalization required for IPD cases. It also incurs significant indirect costs, stemming not only from reduced productivity among parents and caregivers but also from long-term neurological disabilities that can result from post-meningitis sequelae. Collectively, pneumococcal disease places a considerable burden on pediatric patients, their families, healthcare systems, and society as a whole [10]. It is estimated that, without vaccines, PD would account for $13.7 billion in annual health-system costs and $14.3 billion in annual societal costs worldwide. Despite these staggering figures, the true cost burden of PD is likely underestimated due to under-identification of cases [11].

## 2. Available Pneumococcal Vaccines and Their Limitations

PD is considered vaccine-preventable. There are two main types of pneumococcal vaccines: PCVs (pneumococcal conjugate vaccines) and PPSVs (pneumococcal polysaccharide vaccines). PPSVs only use the polysaccharides found on the bacterial capsule to stimulate a T-cell-independent immune response. These polysaccharides have a repetitive structure that allows them to directly activate B cells without the help of T helper cells. This leads to a rapid production of antibodies, mainly of the IgM type. However, since T cells are not involved, long-term immune memory is not generated, and there is no class switching to more effective antibodies like IgG. For this reason, PPSVs are not recommended for children under 2 years of age, as their immune systems are not yet mature enough to respond effectively through this mechanism [12].

Currently available pneumococcal vaccines include PPSV23, PCV10, PCV13, PCV15, PCV20, and PCV21 (as of 1 July 2024) (Table 1).

The first vaccine, called PPSV14, was introduced in the United States in 1977 and protected against 14 serotypes of *Streptococcus pneumoniae*. In 1983, it was replaced by PPSV23, which includes 23 serotypes responsible (1, 2, 3, 4, 5, 6B, 7F, 8, 9N, 9V, 10A, 11A, 12F, 14, 15B, 17F, 18C, 19A, 19F, 20, 22F, 23F, and 33F) for the majority of severe invasive pneumococcal disease cases. Unlike conjugate vaccines, PPSV23 induces a B-cell-mediated immune response without T-cell involvement, making it ineffective in infants and toddlers but effective in older children and adults. PPSV23 is recommended for adults aged ≥65 years and individuals aged 19–64 years with medical conditions that increase the risk of pneumococcal disease. The protection offered by PPSV23 typically wanes within 3 to 5 years in the absence of T cell-mediated support [13]. However, PPSV23 does not reduce nasopharyngeal carriage of *Streptococcus pneumoniae*, meaning it cannot confer herd protection. While it provides broad serotype coverage, its effectiveness in preventing invasive pneumococcal disease (IPD) is lower compared to conjugate vaccines like PCV13. Additionally, PPSV23 does not prevent serotype replacement, a phenomenon where non-vaccine serotypes (NVTs) emerge as predominant causes of disease. In many countries, PPSV23 is used in combination with conjugate vaccines (e.g., PCV13, PCV15, PCV20) to maximize protection against pneumococcal disease. This approach helps address its limitations by leveraging the herd protection benefits of conjugate vaccines. Ongoing surveillance and updated vaccination strategies are essential to optimize its use and address emerging serotypes.

To overcome the limitations of PPSV—especially its ineffectiveness in young children and its inability to generate long-term immune memory—PCVs were developed in 2000, building on the successful model of Hib conjugate vaccines [14]. These vaccines link CPs to carrier proteins such as diphtheria toxoid (PRP-D), meningococcal outer membrane protein (PRP-OMP), CRM197, or tetanus toxoid (PRP-T). By covalently binding the polysaccharides to a protein, the antigens become T-cell-dependent, allowing the immune system to activate helper T cells. This leads to antibody class switching (from IgM to IgG), affinity maturation, and the development of long-lasting immune memory. This transformation significantly improved the immunogenicity of the vaccine, making it effective in both infants and adults, and providing stronger and longer-lasting protection compared to PPSV [15].

Through guidance first issued in 2007, the WHO recommended the inclusion of PCV in national childhood immunization programs (NIPs) worldwide to prevent pneumococcal disease [16]. As of 2020, PCVs have been incorporated into 160 NIPs globally [17].

The first pneumococcal conjugate vaccine developed was the 7-valent pneumococcal conjugate vaccine (PCV7). It was introduced in the late 1990s and early 2000s in the United States and later in Europe in 2001 and targeted seven *Streptococcus pneumoniae* serotypes (4, 6B, 9V, 14, 18C, 19F, and 23F) most commonly responsible for invasive pneumococcal diseases. The introduction of the 7-valent pneumococcal conjugate vaccine (PCV7) in 2000 for infants provided long-lasting immunity and significantly reduced nasopharyngeal carriage of the seven most virulent *Streptococcus pneumoniae* serotypes, leading to the development of herd protection [18]. Following its widespread use, there was a substantial decline in IPDs caused by the serotypes covered by PCV7, particularly among children. Additionally, indirect protection was observed in adults in countries with routine pediatric PCV7 immunization programs.

The 10-valent pneumococcal conjugate vaccine (PCV10) was developed to provide protection against 10 serotypes of *Streptococcus pneumoniae:* 1, 5, 7F, 4, 6B, 9V, 14, 18C, 19F, and 23F. Introduced in 2009, PCV10 has been widely implemented in pediatric NIPs across various countries, demonstrating significant effectiveness in reducing IPD and non-invasive pneumococcal pneumonia in children. PCV10 has shown substantial impact in reducing vaccine-covered serotypes in children, which indirectly benefits adults through herd protection. However, studies have indicated that PCV10 does not provide cross-protection against serotype 19A, a major cause of pneumococcal disease in some regions [19]. This limitation has led several countries to transition to higher-valency vaccines, such as PCV13, which include serotype 19A. In countries where PCV10 remains in use, surveillance data have highlighted the emergence of non-PCV10 serotypes, such as 19A and 3, which contribute to residual pneumococcal disease burden [19]. Despite these challenges, PCV10 continues to play a critical role in reducing pneumococcal disease in regions where it is implemented, particularly in settings with limited access to higher-valency vaccines.

PCV13, the 13-valent pneumococcal conjugate vaccine, was introduced in 2010 to protect against 13 serotypes of *Streptococcus pneumoniae*. These serotypes include the PCV10 serotypes plus 3, 6A, and 19A. The vaccine has been widely incorporated into NIPs in many countries, using dosing schedules such as 2 + 1 or 3 + 1. Its introduction significantly reduced the incidence of IPD and pneumococcal pneumonia in vaccinated children. It also generated herd protection, reducing transmission of the bacteria to unvaccinated adults and children. However, serotype 3, included in PCV13, has shown limited reduction in disease due to its low immunogenicity and ability to evade the immune response [19]. Additionally, as diseases caused by PCV13-covered serotypes decreased, non-vaccine serotypes emerged, such as serotype 8 in Europe and serotype 4 in North America.

WHO data from 2018 show that deaths caused by pneumococcal disease decreased by 51% between 2000 and 2015 following the introduction of conjugate vaccines [20]. Following the introduction of PCV7, PCV10, and PCV13 over the last 20 years, cases of PD and IPD attributable to vaccine serotypes have declined sharply. This has led to reduced morbidity and mortality in children due to these vaccine-type diseases. Global modeling studies estimate that, between 2010 and 2019, PCV13 prevented 175 million cases of pneumococcal disease and 625,000 deaths worldwide in children under five [21].

However, by 2015, a rebound in IPD incidence was observed, primarily due to the emergence of non-PCV13 serotypes. By 2018, these non-vaccine serotypes accounted for a substantial proportion of IPD cases, particularly among older adults. To address this phenomenon, PCV13 was progressively replaced by higher-valency formulations. PCV15 and PCV20 were developed and successively licensed for adult use in 2021, followed by FDA approval of PCV21 in 2024. Despite this, PCV13 remains a milestone in pneumococcal disease prevention and has had a significant impact on global public health.

PCV15, or 15-valent Pneumococcal Conjugate Vaccine, is a vaccine that protects against 15 serotypes of *Streptococcus pneumoniae*. It includes all the serotypes covered by PCV13 (13-valent vaccine) and includes two additional serotypes: 22F and 33F. These serotypes are important because they are associated with invasive pneumococcal diseases, particularly in adults. PCV15 is recommended for use in children, having replaced PCV13 in the pediatric national immunization programs of some countries. It is also recommended for adults, especially those aged 65 years and older or adults between 19 and 64 years with certain underlying medical conditions. PCV15 has demonstrated improved immunogenicity against serotype 3 compared to PCV13, which is significant because serotype 3 is highly virulent and persistent. This vaccine provides broader protection against pneumococcal diseases and is part of efforts to address emerging serotypes not covered by earlier vaccines [15,22]. A 2025 systematic review of its safety found that PCV15 was associated with a higher incidence of local and systemic reactions compared to PCV13. Local reactions, such as redness, swelling, and injection site pain, were more common, with a risk ratio (RR) of 1.23 for any local reactions and 1.26 for injection site pain. Systemic reactions, including myalgia, fatigue, and other mild to moderate adverse events, were also more frequent, with an RR of 1.15. However, no serious adverse events or deaths related to PCV15 were reported [23].

PCV20, or the 20-valent pneumococcal conjugate vaccine, is designed to protect against 20 serotypes of *Streptococcus pneumoniae*. Compared to PCV15, it includes five additional serotypes—8, 10A, 11A, 12F, and 15B—which are associated with invasive pneumococcal disease and help broaden the protective scope of the vaccine. In the pediatric population, PCV20 has replaced PCV13 in the national immunization programs of some countries. For adults, it is particularly recommended for individuals aged 65 and older, as well as for those aged 19 to 64 who have underlying medical conditions [24]. The introduction of PCV20 aims to address both residual and emerging pneumococcal serotypes, especially in populations where herd protection is already well established. As such, it represents a significant advancement in pneumococcal disease prevention strategies. Furthermore, PCV20 showed a slightly higher incidence of local reactions compared to PCV13, with an RR of 1.08. However, systemic reactions were similar between PCV20 and PCV13, with an RR of 1.01. No serious adverse events or unexpected safety concerns were identified for PCV20 [23,25].

The 21-valent pneumococcal conjugate vaccine (PCV21) represents a significant advancement in the prevention of pneumococcal diseases in adults. Approved by the U.S. Food and Drug Administration (FDA) in 2024, PCV21 is specifically designed to address residual and emerging pneumococcal serotypes that continue to cause IPD in adults, particularly in regions with established herd protection. The vaccine includes 21 serotypes: 3, 6A, 7F, 19A, 22F, 33F, 8, 10A, 11A, 12F, 9N, 17F, 20, 15A, 15C, 16F, 23A, 23B, 24F, 31, and 35B [15]. Recent studies have demonstrated that PCV21 provides coverage for 81% of IPD cases in adults aged 16–64 years with risk factors and 85% in adults aged ≥ 65 years [26]. Unlike previous pneumococcal vaccines, such as PCV13, PCV15, and PCV20, PCV21 targets a broader range of serotypes, including those associated with severe disease outcomes, such as serotypes 3, 8, and 19A. PCV21 has been incorporated into adult vaccination recommendations in the United States and Canada. In the U.S., the Advisory Committee on Immunization Practices (ACIP) recommended PCV21 as an option for adults aged ≥18 years for whom pneumococcal vaccination is indicated. Similarly, in Canada, PCV21 was authorized for use in adults aged ≥ 18 years and is recommended for all adults aged ≥65 years and those aged 18–64 years with risk factors for IPD [15]. The introduction of PCV21 is expected to significantly reduce the burden of pneumococcal diseases in adults, particularly in populations at higher risk. However, the inclusion of additional serotypes may alter the epidemiological landscape, necessitating ongoing surveillance to monitor serotype replacement and vaccine effectiveness. Furthermore, the development of PCV21 highlights the need for continued innovation in pneumococcal vaccine strategies to address the limitations of serotype-specific vaccines and the emergence of non-vaccine serotypes.

As foretold, overall impact of PCVs has been limited by the phenomenon of serotype replacement [27], which represents the primary limitation of current polysaccharide-based PCVs [28,29]. By reducing nasopharyngeal colonization of VTs, PCVs exert selective pressure that favors the colonization and expansion of NVTs. The clinical and public health implications of serotype replacement are profound, as emergent NVTs may exhibit increased prevalence, pathogenicity, or antimicrobial resistance compared to the VTs they replace [29,30]. Furthermore, *Streptococcus pneumoniae* can undergo genetic recombination and switch its capsule to an NVT—a process known as capsular switching. The herd effect induced by PCVs can further promote the dominance of NVTs in the population [31]. Moreover, although adding new serotypes to PCVs seems beneficial, this cumulative approach poses certain challenges. Increasing vaccine valency requires larger quantities of carrier protein, which can lead to carrier-induced epitope suppression (CIES)—a phenomenon in which the immune response to individual serotypes is weakened due to competition among T cells recognizing the carrier protein. Moreover, chemical modification of capsular polysaccharides (CPs) during vaccine development can compromise immunogenicity. Activation of CPs for conjugation typically involves oxidation with sodium periodate, a process that can alter their physicochemical properties. This alteration may interfere with antigen presentation through MHC molecules, thus reducing the immune response. Overall, these challenges highlight the urgent need for new conjugation techniques and the development of vaccines targeting conserved structures of *Streptococcus pneumoniae*, capable of offering serotype-independent protection.

## 3. Pneumococcal Vaccines in the Vulnerable

Pneumococcal vaccination is a cornerstone of public health, especially for individuals at high risk of severe infection with Streptococcus pneumoniae, such as those with chronic diseases, asthma, and immunocompromising conditions, where achieving optimal protection is paramount. However, the effectiveness of the vaccine can be affected by health status, disease severity, and treatment regimens, underscoring the need of personalized vaccine strategies that tailored timing and dosing schedules.

### 3.1. Patients with Chronic Diseases

In patients with diabetes mellitus, the risk of pneumococcal infection is significantly elevated. A recent large meta-analysis [32] demonstrated a considerably increased risk of IPD and related severe outcomes, particularly in those with hyperglycemia, insulin resistance, or coinfections. The study found that the pneumococcal conjugate vaccine PCV13 offered robust protective efficacy, with no significant difference observed when compared to the polysaccharide vaccine PPSV23. While newer vaccines like PCV15 and PCV20 are anticipated to be more effective, specific data for people with diabetes are not yet available. Despite potential variability in vaccine effectiveness related to glycemic control, pneumococcal vaccination is associated with reductions in hospitalizations and mortality, although these effects may not always be statistically significant, highlighting the need for broader coverage [33]. A recent expert consensus on adult pneumococcal immunization [34] endorses universal pneumococcal vaccination for all diabetic adults, regardless of glycemic control, emphasizing population-wide immunization as a strategy to reduce complications and healthcare burden.

For patients with cystic fibrosis (CF), vaccination is essential for reducing respiratory tract infections. Data suggest that achieving adequate vaccine coverage in this group remains a challenge. A French multicenter study by Masson et al. [35] investigating 134 CF patients found insufficient coverage rates for mandatory vaccines, including the conjugate pneumococcal vaccine, at one year of age. This low coverage rate is consistent with findings from a recent broader study of children with a spectrum of chronic diseases [36], which reported a PCV coverage rate of 55% and a PPSV coverage rate of only 5.7% among the 366 enrolled patients. Additionally, the immune response to the pneumococcal polysaccharide vaccine has been shown to be impaired in a subgroup of children with CF [37].

Asthma has long been recognized as a condition that increases the risk of pneumococcal disease. The CDC’s Advisory Committee on Immunization Practices [24] first included asthma as an indication for adult pneumococcal vaccination in 2010. Current guidelines [38] endorse the use of PCV15 or PCV20 for adults with asthma, with extended recommendations for older adults. Research by Bhardwaj et al. [39] reported that PCV13 was effective against CAP and IPD in patients with asthma or other chronic lung diseases. While most asthma patients mount a sufficient immune response, the quality of vaccine-induced protection can vary based on the severity and control of the disease.

### 3.2. Immunocompromised Populations

The intersection of immunodeficiency and IPD risk defines a critical area for clinical intervention, demanding highly nuanced and personalized vaccine strategies to mitigate morbidity and mortality. Individuals with compromised immunity, encompassing both Primary Immunodeficiencies (PID) and Secondary Immunodeficiencies (SID), face a significantly elevated incidence of IPD compared to the general population, a vulnerability underscored by systematic review and meta-analysis findings [40]. This predisposition needs a paradigm shift from standard vaccination guidelines toward tailored schedules that account for the underlying immune defect, the degree of immunocompromise, and the response kinetics to different vaccine formulations [41]. The spectrum of high-risk children includes those with PID, asplenia, post-transplantation status, active malignancy, HIV, or iatrogenic Secondary Immunodeficiencies (SID) [42]. Optimal pneumococcal vaccine strategies must therefore prioritize effective prevention while simultaneously leveraging vaccination as a valuable diagnostic tool to characterize and manage underlying immune dysfunction [41].

### 3.3. Rationale for Vaccinating Immunocompromised Children: The Dual Role of Prevention and Diagnosis in Primary Immunodeficiencies

#### 3.3.1. Preventive Role

Children with PID represent a vulnerable cohort, where pneumococcal vaccines can be integral to strategies focused on reducing morbidity and preventing life-threatening infection. The need of rigorous vaccination is highlighted by the high prevalence of underlying immune defects discovered in pediatric patients presenting with IPD [43,44].

Retrospective studies have established that a child presenting with IPD may have an underlying PID that warrants detailed immunological investigation, including assessment for single-gene mutations and B cell dysfunction. Furthermore, children experiencing recurrent IPD (rIPD) may be found to have an underlying condition of immunosuppression, with some reports suggesting the prevalence of an underlying PID or immunosuppressive disorder in those with recurrent IPD episodes can exceed 60% [45], thereby underscoring the importance of early and effective prophylaxis [43,44]. The preventive role of pneumococcal conjugate vaccines is substantiated by clinical data, even in populations with humoral immune defects. A cohort study examining the efficacy of PCV13 in a subset of patients with primary humoral immunodeficiency demonstrated that the vaccine successfully elicited serological protection in approximately 71%, 66% and 56% of patients at one, six and twelve months after immunization [46]. Crucially, the observation that protective antibody responses tended to decline over time in some immunocompromised patients emphasizes the need for tailored revaccination schedules or the implementation of combined vaccine sequences to maintain sustained immunity. Therefore, the core rationale for vaccination is to prevent IPD and other severe pneumococcal infections, directly contributing to a reduction in disease severity and subsequent hospitalization rates. The proactive, timely application of PCV and PPSV to this vulnerable pediatric population thus constitutes an essential pillar of supportive care [42].

#### 3.3.2. Diagnostic Role

Beyond its preventive function, pneumococcal vaccination serves as an essential diagnostic tool in the clinical workup of children—and adults—with recurrent infections or suspected immune dysfunction [41].

In the diagnostic algorithm for antibody deficiencies, the evaluation of specific vaccine response is a core component. Quantitative assessment of serotype-specific IgG titers, both before and 4–8 weeks after vaccination with PPSV23, is the recommended method to evaluate the adequacy of the humoral response and the requirement for more advanced immunological investigations [47]. In this context, the American Academy of Allergy, Asthma & Immunology (AAAAI) established guidelines that advocate measuring antibodies against multiple pneumococcal CPs serotypes both before and four to eight weeks after administering the 23-valent pneumococcal polysaccharide vaccine. The current assessment criteria rely on a dichotomous threshold, classifying a response as protective if a specific number of anti- CPs antibodies achieve or overcome a post-vaccine concentration of 1.3 mg/L [41]. Moreover, the presence of a suboptimal specific antibody response is considered for treatment decisions (i.e., Immunoglobulin Replacement Therapy, IGRT).

The evaluation of antibody response to PPSV23 is the gold standard to diagnose Specific Antibody Deficiency (SAD), a humoral defect characterized by impaired antibody production against polysaccharide antigens despite otherwise normal immunoglobulin levels [47].

Apart from SAD, a growing subset of monogenic Inborn Errors of Immunity (IEI) (e.g., NFKB1 Deficiency, NFKB2 Deficiency, CTLA4 deficiency, Activated PI3K δ Syndrome APDS), may solely start with an impairment of the IgG response to polysaccharide antigens, despite otherwise preserved serum levels of total IgG and intact antibody responses to T-cell-dependent antigens, such as protein antigens (e.g., tetanus toxoid) or polysaccharide-conjugate vaccines. A quantitative assessment of anti-polysaccharide IgG antibodies is recommended for the diagnosis of several rare IEIs. A protracted diagnostic interval inevitably postpones critical therapeutic interventions, potentially resulting in chronic infectious morbidity and organ damage (e.g., bronchiectasis) [48].

Recent developments in pneumococcal vaccination, particularly the increasing use of higher-valency conjugate vaccines in pediatric and adult populations, are progressively reducing the reliance on PPSV23 in routine immunization schedules. This shift, while beneficial for longer lasting protective coverage, limits T-cell-independent antibody responses assessment.

Current interpretative guidelines from the AAAAI suffer from significant drawbacks. First, the criteria, which rely on a fixed, dichotomous numerical threshold of 1.3 mg/L for post-vaccination antibodies, have proven overly sensitive. The standard was historically established by expert consensus rather than validated by robust, data-driven models. This intrinsic flaw results in an alarmingly high false positive rate, with studies indicating that a significant fraction of presumed healthy, immunocompetent individuals—ranging between 20% and 60%—may be erroneously classified as antibody-deficient. Such misdiagnosis leads to undue patient anxiety, the risk of unnecessary treatment exposure, and substantial resource utilization. Thus, the potential future discontinuation of PPSV23 necessitates the adoption of more innovative functional testing methods and alternative diagnostic strategies to bridge the gap.

Second, the methodology is complicated by technological evolution. The transition from the older ELISA technique to modern multianalyte bead-based assays (multiplex assays) introduces inter-assay variability and compromises the direct applicability of the original, ELISA-derived 1.3 mg/L threshold [49].

Moreover, further interpretative challenges are age-dependent responses and potential interference from prior vaccination history or concurrent IGRT [47].

Of note, adding to these challenges is the widespread adoption of pneumococcal conjugate vaccines (PCVs) such as PCV13, PCV15 and PCV20 in both children and adults [50,51].

To overcome the shortcomings of fixed thresholds, the Polysaccharide Responsiveness Percentile (PRP) method is emerging as a robust, non-parametric paradigm. This streamlined approach minimizes complexity by utilizing only a single post-vaccination sample, eliminating the need for a baseline measurement. The core of the PRP is to compare the patient’s individual serotype-specific antibody levels against the percentile ranks of a healthy, vaccinated reference population. By aggregating these percentiles, the final PRP score offers a highly nuanced, clinically interpretable measure of immune function that is naturally adjusted for serotype immunogenicity differences and less susceptible to the methodological fragility of fixed cutoffs [49].

Among the alternative diagnostic approaches to assess T cell independent antibody response, the use of other pure polysaccharide vaccines, such as the Salmonella typhi Vi polysaccharide vaccine [52], may be considered.

The role of surrogate markers such as isohemagglutinin titers has also been explored, though with limited concordance and requirement of further validation studies [53].

While not standard practice for routine screening, the assay of IgM and IgA class antibodies specific to pneumococcal polysaccharides—both pre- and post-vaccination—can provide supplementary diagnostic utility. These specialized tests become particularly valuable when assessing patients with IEI who are already receiving IGRT, as the concurrent presence of exogenous IgG compromises the accurate measurement of the endogenous IgG response [48].

Studies investigating the total IgM, IgA, and IgG antibody responses to PPSV23 in healthy adults and PID patients have contributed important data to better defining normal reference ranges and understanding the complex kinetics of the non-IgG isotypes following vaccination [54]. The continued refinement of these assays to enhance the sensitivity and specificity of diagnostic vaccination paves its role as a fundamental tool to characterize the specific antibody response in vulnerable patients.

### 3.4. Personalized Vaccine Schedules in Secondary Immunodeficiencies: Optimizing the Immunological Window

In patients with SID, such as those with solid organ or hematopoietic stem cell transplants or those receiving systemic immunosuppressive agents for autoimmune diseases or malignancy, the timing of vaccination is paramount to maximize immune response [41,42]. Current expert recommendations emphasize that pneumococcal vaccination should ideally be administered before the start of immunosuppressive treatments [41,55]. This strategy leverages the temporary preservation of immunological competence prior to iatrogenic blunting of the adaptive immune system, allowing the patient to mount the strongest possible T-cell-dependent and T-cell-independent antibody response. Data concerning the immunological response to pneumococcal vaccines in SID adults often suggest that the efficacy, particularly against the PPSV23, is both diminished and short-lived [56]. This compromised duration and magnitude of protection mandates the use of synergistic vaccine strategies including the adoption of higher-valency PCVs like PCV20 and PCV21, which offer broader serotype coverage against emerging non-vaccine types prevalent in immunocompromised adults. PCV20 has demonstrated robust immunogenicity in adults aged 18–64 years with medical risk conditions, eliciting strong opsonophagocytic responses across its 20 serotypes, including those associated with residual IPD burden post-PCV13 [57]. Similarly, the 2024 ACIP recommendations endorse PCV21 as single-dose option for adults ≥19 years with immunocompromising condition and other risk indications who are currently recommended to receive PCV15 or PCV20, covering eight unique serotypes responsible for ~20–30% of IPD in at risk groups and providing non inferior responses to PCV20 for shared serotypes [58]. A comprehensive review of pneumococcal vaccination strategies in immunocompromised adults, particularly those undergoing chemotherapy or in the post-transplantation phase, strongly advocates for a combined approach utilizing both the protein-conjugate and polysaccharide vaccines [59]. The preferred strategy involves the sequential administration of PCV13 followed by PPSV23. The rationale for this PCV13-to-PPSV23 sequence is rooted in the T-cell-dependent nature of the conjugate vaccine, which is capable of inducing immunological memory and generating a more robust, longer-lasting, and higher-affinity IgG antibody response against the shared serotypes [41]. Subsequently, the PPSV23 booster provides broader coverage against additional pneumococcal serotypes via a T cell independent mechanism. This sequential immunization aims to elicit a stronger and broader overall protective immune response in this highly susceptible population. The combined use of these vaccines is a cornerstone of personalized vaccine schedules for immunocompromised adults, to achieve high levels of durable immune protection [59].

## 4. Future Perspectives and Implications for Clinical Practice and Public Health

Despite the undeniable successes of PCVs global implementation, the continued evolution of *Streptococcus pneumoniae* and the looming crisis of antimicrobial resistance (AMR) necessitate a clear articulation of future perspectives, focusing on the sustained impact of current vaccination strategies, the critical need for dynamic surveillance, and the pursuit of next-generation, serotype-independent vaccines [28,29]. The future of pneumococcal disease prevention and the mitigation of associated AMR depend on coordinated global action across these three interconnected domains.

### 4.1. Impact of PCV Vaccination on Bacterial Antimicrobial Resistance

Pneumococcal vaccination is recognized as a key preventive measure in the WHO Global Action Plan on AMR, acting through a dual mechanism to reduce the prevalence of resistant infections and subsequent antibiotic use [60,61]. The primary mechanism involves the direct reduction of the incidence of vaccine-type (VT) disease and of antibiotic treatment [62]. The secondary, yet equally critical, effect is the indirect reduction of antibiotic resistant infections through herd protection and decreased carriage of antibiotic resistant strains [62]. This preventive strategy averts the selective pressure that drives resistance, marking a proactive and cost-effective approach to AMR containment [61]. The economic and public health value of this intervention is substantial, though historically under-quantified [61]. Modeling studies, such as those using the DREAMR platform, have begun to quantify this value, demonstrating that the introduction of PCV vaccination has slowed down the development of AMR to key antibiotics [62]. For instance, PCV introduction in Ethiopia was estimated to reduce the development of resistance to amoxicillin by approximately 14.77% and, critically, to have averted around 718,100 antibiotic treatment failures and 9520 AMR-related deaths between 2011 and 2017, representing a nearly 28% reduction in AMR-related fatalities [62]. This underscores the extensive benefit of PCVs beyond disease prevention and their position as essential tools in the fight against AMR [62]. Furthermore, vaccination is now included in a high percentage of national AMR action plans with growing recognition by WHO Member States of its importance as a core pillar of preventative strategy [60]. However, the efficacy of PCV in combating AMR is challenged by the phenomenon of serotype replacement, which shifts the burden of resistance to Non-Vaccine Serotypes (NVTs) [30,61,63]. While PCV introduction leads to a marked decrease in the carriage of resistant VT strains, some studies have observed an increase in the proportion of antibiotic-resistant NVTs [30,61,63]. Surveillance data from Portugal post-PCV13 introduction showed a significant reduction in carriage prevalence for PCV13 serotypes (dropping from 47.6% pre-vaccine to 10.7% post-vaccine), including resistant strains like 19F, 3, and 19A [63]. Despite this success, the overall pneumococcal carriage prevalence remained stable, indicating the dominance of NVTs and the necessity to monitor their resistance profiles [63]. Similarly, in Spain, national surveillance demonstrated a significant decrease in penicillin and macrolide resistance among pneumococcal isolates following PCV introduction, but this benefit was attenuated by the emergence of resistant serotypes not covered by the vaccine [30]. The COVID-19 pandemic further complicated this landscape, with studies in Spain reporting an emergence of serotypes with reduced susceptibility during the initial year of the pandemic, emphasizing the fragility of gains against AMR in the context of major public health crises and altered antibiotic prescribing [30]. Longitudinal studies in tertiary care centers in regions like India, where PCV was recently introduced, are actively monitoring the expected shift in both serotype and AMR patterns, confirming that resistance patterns are intrinsically linked to serotype ecology [64]. Therefore, future clinical practice must integrate antibiotic stewardship with vaccine programs, recognizing that the long-term success of PCVs in mitigating AMR hinges on continuous epidemiological surveillance to quickly identify and respond to resistant NVT emergence [30,61,63].

### 4.2. Importance of Emergent Serotype Identification to Develop New Vaccine Formulations

Continued surveillance and emergent serotype identification are thus paramount to inform vaccine development and optimize vaccination strategies [29]. The epidemiology of emerging and spreading NVTs must be the central focus of future serotype epidemiological surveys and vaccine optimization efforts [29].

After the introduction of PCV7, numerous studies reported an increase in infections caused by non-vaccine serotypes, particularly serotype 19A. Countries adopting sustained use of PCV13 in NIPs have observed an increased incidence of multiple non-PCV13 serotypes, including, but not limited to serotypes 6C, 8, 9N, 10A, 11A, 12F, 15A, 15B, 15C, 22F, 23B, 24F, 33F, 35B, and 38. Even though the non-vaccine serotypes are generally less pathogenic and the overall rates of IPD remain substantially lower than pre-vaccine levels, some serotypes have started to become endemic or cause outbreaks [65,66].

To reduce the remaining pediatric IPD burden, these serotypes have become the focus of expanded-valency PCVs licensed in more recent years: PCV15 [PCV13 serotypes plus 22F and 33F] and PCV20 [PCV15 serotypes plus 8, 10A, 11A, 12F, and 15B] [67]. Of interest, a review of 118 studies conducted in 33 European countries (2010–2022) found that serotypes unique to PCV20 (8, 10A, 11A, 12F, 15B, 22F, and 33F) have become increasingly prevalent in adults with both invasive and non-invasive pneumococcal disease. These serotypes are often associated with greater disease severity, mortality, and antimicrobial resistance, particularly affecting the elderly and high-risk individuals. Since 2018–2019, they have accounted for approximately 60% of adult IPD cases in Europe. Serotype replacement varies by region and population and remains a key driver behind efforts to develop higher-valency and serotype-independent vaccine strategies.

One particularly relevant emergent serotype is 6C, which represents a substantial burden of pneumococcal disease globally [68]. Serotype 6C is a derivative of 6A but is not directly included in PCV10 (which contains 6B) or PCV13 (which contains 6A and 6B) [68]. The same is true for PCV15, PCV20 and PCV21. While some studies suggest that PCV13 may confer cross-protection against 6C invasive pneumococcal disease, particularly with increasing vaccination coverage, the data remain complex and insufficient to definitively determine the effectiveness of PCV10 or PCV13 against 6C carriage and AMR [68].

This ambiguity highlights a critical area for future research and vaccine refinement: serotypes with complex capsular structures or those generated through capsular switching demand enhanced surveillance and targeted inclusion in next-generation formulations [28,29]. Optimizing vaccination strategies means leveraging genomic surveillance alongside traditional serotyping [30]. Genomic studies can provide granular insights into the lineages associated with serotype replacement and antibiotic resistance, allowing for a more nuanced understanding of the ecological shift in the pneumococcal population [30]. The ultimate goal of this sustained surveillance is to provide the empirical data necessary for policymakers to periodically update national immunization programs by adopting higher-valent PCVs or, more fundamentally, by transitioning to vaccines designed for universal protection [29].

### 4.3. Protein Candidates for a Universal Pneumococcal Vaccine

The persistent threat of serotype replacement and the rise of non-encapsulated *Streptococcus pneumoniae* strains have fueled the search for a truly serotype-independent, or “universal,” pneumococcal vaccine. This novel strategy focuses on conserved pneumococcal surface proteins (PSPs) rather than the variable capsular polysaccharides that define current vaccines [28]. The potential of protein-based vaccines to overcome the inherent limitations of current polysaccharide-based vaccines lies in their ability to offer broader serotype coverage and target proteins essential for bacterial survival, regardless of the capsular type [28]. Several key pneumococcal surface proteins are being aggressively investigated as candidates for universal vaccine formulations due to their high conservation across diverse pneumococcal serotypes and their critical roles in bacterial pathogenesis, including adhesion, immune evasion, and biofilm formation [28].

Pneumolysin (Ply): a potent, pore-forming toxin that is critical for pneumococcal virulence and highly conserved Due to its toxicity, advances in protein engineering have focused on developing detoxified derivatives (toxoids, such as PiuA-PlyD4) that retain high immunogenicity while eliminating the cytolytic activity.

Choline-Binding Proteins (CBPs): this family of surface proteins is defined by a conserved choline-binding domain (CBD) that anchors them to the bacterial surface. Key candidates within this group include Pneumococcal Surface Protein A (PspA) and Pneumococcal Surface Protein C (PspC). PspA is crucial for immune evasion, and while its N-terminal domain is structurally variable, it contains conserved regions that can elicit protective immune responses. PspC is likewise involved in adhesion and complement interference.

Histidine Triad Proteins (PHTS): this family, which includes PhtD, is involved in metal acquisition, with candidates like PhtD demonstrating potential in multivalent formulations.

The future for protein-based pneumococcal vaccines centers on multivalent strategies—combining two or more conserved proteins to achieve robust, non-overlapping protection [28,29].

Preclinical studies have shown promise for multivalent formulations incorporating antigens like PhtD and PspA, highlighting the feasibility of a pan-pneumococcal vaccine [69]. Such vaccines are designed not only to prevent IPD but also to limit nasopharyngeal carriage, thereby interrupting transmission and providing indirect protection (herd effect) that would be serotype-independent, effectively closing the loop hole exploited by serotype replacement [28]. Novel technologies, including reverse vaccinology and extracellular vesicle (EV)-based vaccine platforms, are further accelerating the discovery and optimization of these candidates. Revolutionizing the field of vaccine science, Reverse Vaccinology (RV) involves the systematic use of genomic and proteomic information as the basis for discovering and selecting candidate vaccines [70,71].

These technological advancements are pivotal to moving beyond the current polysaccharide-based paradigm and implementing a transformative approach to global pneumococcal disease prevention that is capable of mitigating the rise of antibiotic-resistant pneumococcal strains on a global scale [28].

## 5. Conclusions

The successful global implementation of pneumococcal conjugate vaccines (PCVs) has fundamentally shifted the epidemiology of *Streptococcus pneumoniae*, achieving substantial reductions in vaccine-type invasive disease and key indirect benefits such as herd protection and a reduction in AMR. However, the phenomenon of serotype replacement by non-vaccine serotypes remains a significant challenge that limits the overall long-term impact of current polysaccharide-based PCVs. Addressing this reality necessitates continuous, high-resolution epidemiological surveillance and genomic monitoring to inform timely vaccine composition updates. Looking forward, the development of novel, universal pneumococcal vaccines represents a promising strategy to overcome the limitations of serotype-specific vaccines and provide broad, serotype-independent and durable protection.

Ultimately, an integrated global strategy—combining universal vaccination technology, precision public health measures for high-risk populations, and robust antibiotic stewardship—is essential to sustain and expand upon the historic achievements of PCVs, ensuring future generations are protected from both pneumococcal disease and the escalating crisis of global AMR.

## Figures and Tables

**Table 1:** Currently available pneumococcal vaccines.

Type	Adjuvant	Protein Carrier	Serotypes
**PPSV23**	None	None, polysaccharide	1, 2, 3, 4, 5, 6B, 7F, 8, 9N, 9V, 10A, 11A, 12F, 14, 15B, 17F, 18C, 19F, 20, 22F, 23F, and 33F
**PCV10**	Aluminium phosphate	Non-toxic diphtheria CRM197	1, 5, 6A, 6B, 7F, 9V, 14, 19A, 19F and 23F
**PCV13**	Aluminium phosphate	Non-toxic diphtheria CRM197	1, 3, 4, 5, 6A, 6B, 7F, 9V, 14, 18C, 19A, 19F and 23F
**PCV15**	Aluminium phosphate	Non-toxic diphtheria CRM197	1, 3, 4, 5, 6A, 6B, 7F, 9V, 14, 18C, 19A, 19F, 22F, 23F and 33F
**PCV20**	Aluminium phosphate	Non-toxic diphtheria CRM197	1, 3, 4, 5, 6A, 6B, 7F, 8, 9V, 10A, 11A, 12F, 14, 15B, 18C, 19A, 19F, 22F, 23F and 33F
**PCV21**	none	Non-toxic diphtheria CRM197	3, 6A, 7F, 19A, 22F, 33F, 8, 10A, 11A, 12F, 9N, 17F, 20, 15A, 15C, 16F, 23A, 23B, 24F, 31, and 35B

PCV, pneumococcal conjugate vaccine; PPSV, pneumococcal polysaccharides vaccine.

## Data Availability

No new data were created or analyzed in this study. Data sharing is not applicable to this article.

## Abbreviations

The following abbreviations are used in this manuscript:

IPD	Invasive Pneumococcal Disease
PCVs	Pneumococcal Conjugate Vaccines
CPs	capsular polysaccharides
CAP	community-acquired pneumonia
AOM	acute otitis media
SAARC	South Asian Association for Regional Cooperation
PD	Pneumococcal disease
NHICs	non-high-income countries
WHO	World Health Organization
US	United States
PPSVs	pneumococcal polysaccharide vaccines
NVTs	non-vaccine serotypes
NIPs	national immunization programs
RR	risk ratio
ACIP	Advisory Committee on Immunization Practices
CIES	carrier-induced epitope suppression
CF	cystic fibrosis
PID	Primary Immunodeficiencies
SID	Secondary Immunodeficiencies
AAAAI	American Academy of Allergy, Asthma & Immunology
PRP	Polysaccharide Responsiveness Percentile
IEI	Inborn Errors of Immunity
IGRT	Immunoglobulin Replacement Therapy
AMR	antimicrobial resistance
VT	vaccine-type
PSPs	pneumococcal surface proteins
CBPs	Choline-Binding Proteins
CBD	choline-binding domain
PspA	Pneumococcal Surface Protein A
PspC	Pneumococcal Surface Protein C
PHTS	Histidine Triad Proteins
EV	extracellular vesicle
RV	Reverse Vaccinology
FDA	Food and Drug Administration
APDS	Activated PI3K δ Syndrome

## References

[1] Narciso A.R., Dookie R., Nannapaneni P., Normark S., Henriques-Normark B. (2025). Streptococcus pneumoniae epidemiology, pathogenesis and control. Nat. Rev. Microbiol..

[2] Weiser J.N., Ferreira D.M., Paton J.C. (2018). Streptococcus pneumoniae: Transmission, colonization and invasion. Nat. Rev. Microbiol..

[3] Izurieta P., AbdelGhany M., Borys D. (2025). Serotype distribution of invasive and non-invasive pneumococcal disease in children ≤5 years of age following the introduction of 10-and 13-valent pneumococcal conjugate vaccines in infant national immunization programs: A systematic literature review. Front. Public Health.

[4] Cui Y.A., Patel H., O’Neil W.M., Li S., Saddier P. (2017). Pneumococcal serotype distribution: A snapshot of recent data in pediatric and adult populations around the world. Hum. Vaccines Immunother..

[5] Jaiswal N., Singh M., Das R.R., Jindal I., Agarwal A., Thumburu K.K., Kumar A., Chauhan A. (2014). Distribution of serotypes, vaccine coverage, and antimicrobial susceptibility pattern of Streptococcus pneumoniae in children living in SAARC countries: A systematic review. PLoS ONE.

[6] European Centre for Disease Prevention and Control (ECDC) Invasive Pneumococcal Disease—Annual Epidemiological Report for 2022. https://www.ecdc.europa.eu/en/publications-data/invasive-pneumococcal-disease-annual-epidemiological-report-2022.

[7] Weaver J., Hu T., Podmore B., Barnett R., Obermüller D., Galetzka W., Qizilbash N., Haeckl D., Weiss T., Mohanty S. (2024). Incidence of pneumococcal disease in children in Germany, 2014–2019: A retrospective cohort study. BMC Pediatr..

[8] Kolhapure S., Yewale V., Agrawal A., Krishnappa P., Soumahoro L. (2021). Invasive Pneumococcal Disease burden and PCV coverage in children under five in Southeast Asia: Implications for India. J. Infect. Dev. Ctries..

[9] Kaur R., Morris M., Pichichero M.E. (2017). Epidemiology of acute otitis media in the postpneumococcal conjugate vaccine era. Pediatrics.

[10] Huang M., Xie J., Romdhani H., Song Y., Lee S., Liu D., Elbasha E., Mohanty S., Rowen D., Kelly M.S. (2025). Global assessment of health utilities associated with pneumococcal disease in children—Targeted literature reviews. PharmacoEconomics.

[11] Syeed M.S., Ghule P., Le L.M., Veettil S.K., Horn E.K., Perdrizet J., Wasserman M., Thakkinstian A., Chaiyakunapruk N. (2023). Pneumococcal vaccination in children: A systematic review and meta-analysis of cost-effectiveness studies. Value Health.

[12] Laferriere C. (2011). The immunogenicity of pneumococcal polysaccharides in infants and children: A meta-regression. Vaccine.

[13] Diao W.Q., Shen N., Yu P.X., Liu B.B., He B. (2016). Efficacy of 23-valent pneumococcal polysaccharide vaccine in preventing community-acquired pneumonia among immunocompetent adults: A systematic review and meta-analysis of randomized trials. Vaccine.

[14] Ozisik L. (2025). The New Era of Pneumococcal Vaccination in Adults: What Is Next?. Vaccines.

[15] Morimoto K., Masuda S. (2025). Pneumococcal vaccines for prevention of adult pneumonia. Respir. Investig..

[16] Deloria Knoll M., Bennett J.C., Garcia Quesada M., Kagucia E.W., Peterson M.E., Feikin D.R., Cohen A.L., Hetrich M.K., Yang Y., Sinkevitch J.N. (2021). Global landscape review of serotype-specific invasive pneumococcal disease surveillance among countries using PCV10/13: The pneumococcal serotype replacement and distribution estimation (PSERENADE) project. Microorganisms.

[17] Horn E.K., Wasserman M.D., Hall-Murray C., Sings H.L., Chapman R., Farkouh R.A. (2021). Public health impact of pneumococcal conjugate vaccination: A review of measurement challenges. Expert Rev. Vaccines.

[18] Azzari C., Cortimiglia M., Nieddu F., Moriondo M., Indolfi G., Mattei R., Zuliani M., Adriani B., Degl’Innocenti R., Consales G. (2016). Pneumococcal serotype distribution in adults with invasive disease and in carrier children in Italy: Should we expect herd protection of adults through infants’ vaccination?. Hum. Vaccines Immunother..

[19] Bennett J.C., Deloria Knoll M., Kagucia E.W., Garcia Quesada M., Zeger S.L., Hetrich M.K., Yang Y., Herbert C., Ogyu A., Cohen A.L. (2025). Global impact of ten-valent and 13-valent pneumococcal conjugate vaccines on invasive pneumococcal disease in all ages (the PSERENADE project): A global surveillance analysis. Lancet Infect. Dis..

[20] Wahl B., O’Brien K.L., Greenbaum A., Majumder A., Liu L., Chu Y., Lukšić I., Nair H., McAllister D.A., Campbell H. (2018). Burden of Streptococcus pneumoniae and Haemophilus influenzae type b disease in children in the era of conjugate vaccines: Global, regional, and national estimates for 2000–15. Lancet Glob. Health.

[21] Chapman R., Sutton K., Dillon-Murphy D., Patel S., Hilton B., Farkouh R., Wasserman M. (2020). Ten year public health impact of 13-valent pneumococcal conjugate vaccination in infants: A modelling analysis. Vaccine.

[22] Platt H.L., Cardona J.F., Haranaka M., Schwartz H.I., Perez S.N., Dowell A., Chang C.-J., Dagan R., Tamms G.M., Sterling T. (2022). A phase 3 trial of safety, tolerability, and immunogenicity of V114, 15-valent pneumococcal conjugate vaccine, compared with 13-valent pneumococcal conjugate vaccine in adults 50 years of age and older (PNEU-AGE). Vaccine.

[23] Ngamprasertchai T., Ruenroengbun N., Kajeekul R. (2025). Immunogenicity and safety of the higher-valent pneumococcal conjugate vaccine vs the 13-valent pneumococcal conjugate vaccine in older adults: A systematic review and meta-analysis of randomized controlled trials. Infect. Dis..

[24] Kobayashi M., Pilishvili T., Farrar J.L., Leidner A.J., Gierke R., Prasad N., Moro P., Campos-Outcalt D., Morgan R.L., Long S.S. (2023). Pneumococcal Vaccine for Adults Aged ≥19 Years: Recommendations. MMWR Recomm. Rep..

[25] Klein N.P., Peyrani P., Yacisin K., Caldwell N., Xu X., Scully I.L., Scott D.A., Jansen K.U., Gruber W.C., Watson W. (2021). A phase 3, randomized, double-blind study to evaluate the immunogenicity and safety of 3 lots of 20-valent pneumococcal conjugate vaccine in pneumococcal vaccine-naive adults 18 through 49 years of age. Vaccine.

[26] Yonts A.B., Gaviria-Agudelo C., Kimberlin D.W., Paulsen G.C., O’Leary S.T. (2024). June 2024 ACIP Meeting Update: Influenza, COVID-19, RSV, and Other Vaccines. Pediatrics.

[27] Sari R.F., Fadilah F., Maladan Y., Sarassari R., Safari D. (2024). A narrative review of genomic characteristics, serotype, immunogenicity, and vaccine development of Streptococcus pneumoniae capsular polysaccharide. Clin. Exp. Vaccine Res..

[28] Gopalakrishnan S., Jayapal P., John J. (2025). Pneumococcal surface proteins as targets for next-generation vaccines: Addressing the challenges of serotype variation. Diagn. Microbiol. Infect. Dis..

[29] Du Q.-q., Shi W., Yu D., Yao K.-h. (2021). Epidemiology of non-vaccine serotypes of Streptococcus pneumoniae before and after universal administration of pneumococcal conjugate vaccines. Hum. Vaccines Immunother..

[30] Sempere J., Llamosí M., López Ruiz B., Del Río I., Pérez-García C., Lago D., Gimeno M., Coronel P., González-Camacho F., Domenech M. (2022). Effect of pneumococcal conjugate vaccines and SARS-CoV-2 on antimicrobial resistance and the emergence of Streptococcus pneumoniae serotypes with reduced susceptibility in Spain, 2004–2020: A national surveillance study. Lancet Microbe.

[31] Naucler P., Galanis I., Petropoulos A., Granath F., Morfeldt E., Örtqvist Å., Henriques-Normark B. (2022). Chronic disease and immunosuppression increase the risk for nonvaccine serotype pneumococcal disease: A nationwide population-based study. Clin. Infect. Dis..

[32] Silverii G.A., Gabutti G., Tafuri S., Sarti F., Pratesi A., Clerico A., Fornengo R., Greco C., Irace C., Sordi V. (2024). Diabetes as a risk factor for pneumococcal disease and severe related outcomes and efficacy/effectiveness of vaccination in diabetic population. Results from meta-analysis of observational studies. Acta Diabetol..

[33] Del Riccio M., Boccalini S., Cosma C., Vaccaro G., Bonito B., Zanella B., Salvati C., Giorgetti D., Rigon L., Biamonte M.A. (2023). Effectiveness of pneumococcal vaccination on hospitalization and death in the adult and older adult diabetic population: A systematic review. Expert Rev. Vaccines.

[34] Koul P.A., Vora A.C., Jindal S.K., Ramasubramanian V., Narayanan V., Tripathi S.K., Bahera D., Chandrashekhar H.B., Mehta R., Raval N. (2024). Expert panel opinion on adult pneumococcal vaccination in the post-COVID era. Lung India.

[35] Masson A., Launay O., Delaisi B., Bassinet L., Remus N., Lebourgeois M., Chedevergne F., Bailly C., Foucaud P., Corvol H. (2015). Vaccine coverage in CF children: A French multicenter study. J. Cyst. Fibros..

[36] Yırgın K., Gür E., Erener-Ercan T., Can G. (2023). Vaccination Status in Children with Chronic Diseases: Are They Up-to-Date for Mandatory and Specific Vaccines?. Turk. Arch. Pediatr..

[37] Browning M.J., Lim M.T., Kenia P., Whittle M., Doffinger R., Barcenas-Morales G., Kumararatne D., Viskaduraki M., O'CAllaghan C., Gaillard E.A. (2014). Pneumococcal polysaccharide vaccine responses are impaired in a subgroup of children with cystic fibrosis. J. Cyst. Fibros..

[38] Kobayashi M., Leidner A.J., Gierke R., Xing W., Accorsi E., Moro P., Kamboj M., Kuchel G.A., Schechter R., Loehr J. (2025). Expanded Recommendations for Use of Pneumococcal Conjugate Vaccines Among Adults Aged ≥50 Years: Recommendations of the Advisory Committee on Immunization Practices—United States, 2024. MMWR Morb. Mortal. Wkly. Rep..

[39] Bhardwaj P., Dhar R., Khullar D., Rath P., Swaminathan S., Tiwaskar M., Kulkarni N., Choudhari S., Taur S. (2025). Role of Pneumococcal Vaccination as a Preventative Measure for At-risk and High-risk Adults: An Indian Narrative. J. Assoc. Physicians India.

[40] van Aalst M., Lötsch F., Spijker R., van der Meer J.T.M., Langendam M.W., Goorhuis A., Grobusch M.P., de Bree G.J. (2018). Incidence of invasive pneumococcal disease in immunocompromised patients: A systematic review and meta-analysis. Travel Med. Infect. Dis..

[41] Orange J.S., Ballow M., Stiehm E.R., Ballas Z.K., Chinen J., De La Morena M., Kumararatne D., Harville T.O., Hesterberg P., Koleilat M. (2012). Use and interpretation of diagnostic vaccination in primary immunodeficiency: A working group report of the Basic and Clinical Immunology Interest Section of the American Academy of Allergy, Asthma & Immunology. J. Allergy Clin. Immunol..

[42] van Warmerdam J., Campigotto A., Bitnun A., MacDougall G., Kirby-Allen M., Papsin B., McGeer A., Allen U., Morris S.K. (2023). Invasive Pneumococcal Disease in High-risk Children: A 10-Year Retrospective Study. Pediatr. Infect. Dis. J..

[43] Ingels H., Schejbel L., Lundstedt A.C., Jensen L., Laursen I.A., Ryder L.P., Heegaard N.H., Konradsen H., Christensen J.J., Heilmann C. (2015). Immunodeficiency among children with recurrent invasive pneumococcal disease. Pediatr. Infect. Dis. J..

[44] Gaschignard J., Levy C., Chrabieh M., Boisson B., Bost-Bru C., Dauger S., Dubos F., Durand P., Gaudelus J., Gendrel D. (2014). Invasive pneumococcal disease in children can reveal a primary immunodeficiency. Clin. Infect. Dis..

[45] Bertran M., Abdullahi F., D’Aeth J.C., Amin-Chowdhury Z., Andrews N.J., Eletu S., Litt D., Ramsay M.E., Oligbu G., Ladhani S.N. (2025). Recurrent invasive pneumococcal disease in children: A retrospective cohort study, England, 2006/07-2017/18. J. Infect..

[46] Robbins A., Bahuaud M., Hentzien M., Maestraggi Q., Barbe C., Giusti D., Le Naour R., Batteux F., Servettaz A. (2021). The 13-Valent Pneumococcal Conjugate Vaccine Elicits Serological Response and Lasting Protection in Selected Patients With Primary Humoral Immunodeficiency. Front. Immunol..

[47] Peterson L.K. (2022). Application of vaccine response in the evaluation of patients with suspected B-cell immunodeficiency: Assessment of responses and challenges with interpretation. J. Immunol. Methods.

[48] Fasshauer M., Dinges S., Staudacher O., Völler M., Stittrich A., von Bernuth H., Wahn V., Krüger R. (2024). Monogenic Inborn Errors of Immunity with impaired IgG response to polysaccharide antigens but normal IgG levels and normal IgG response to protein antigens. Front. Pediatr..

[49] Fogsgaard S.F., Todaro S., Larsen C.S., Jørgensen C.S., Jensen J.M.B. (2025). Reassessing Polysaccharide Responsiveness: Unveiling Limitations of Current Guidelines and Introducing the Polysaccharide Responsiveness Percentile Approach. J. Clin. Immunol..

[50] Ameratunga R., Longhurst H., Leung E., Steele R., Lehnert K., Woon S.T. (2024). Limitations in the clinical utility of vaccine challenge responses in the evaluation of primary antibody deficiency including Common Variable Immunodeficiency Disorders. Clin. Immunol..

[51] Zuzolo J., Zulfiqar M.F., Spoelhof B., Revell R., Patrie J.T., Borish L., Lawrence M.G. (2025). Functional testing of humoral immunity in the Prevnar 20 era. Ann. Allergy Asthma Immunol..

[52] Substitutes for Pneumovax to Determine Anti-Polysaccharide IgG Responses. https://www.aaaai.org/allergist-resources/ask-the-expert/answers/2022/pneumovax.

[53] Perazzio S.F., Palmeira P., Moraes-Vasconcelos D., Rangel-Santos A., de Oliveira J.B., Andrade L.E.C., Carneiro-Sampaio M. (2021). A Critical Review on the Standardization and Quality Assessment of Nonfunctional Laboratory Tests Frequently Used to Identify Inborn Errors of Immunity. Front. Immunol..

[54] Parker A.R., Park M.A., Harding S., Abraham R.S. (2019). The total IgM, IgA and IgG antibody responses to pneumococcal polysaccharide vaccination (Pneumovax®23) in a healthy adult population and patients diagnosed with primary immunodeficiencies. Vaccine.

[55] Martire B., Ottaviano G., Sangerardi M., Sgrulletti M., Chini L., Dellepiane R.M., Montin D., Rizzo C., Pignata C., Marseglia G.L. (2022). Vaccinations in Children and Adolescents Treated With Immune-Modifying Biologics: Update and Current Developments. J. Allergy Clin. Immunol. Pract..

[56] Dendle C., Stuart R.L., Mulley W.R., Holdsworth S.R. (2018). Pneumococcal vaccination in adult solid organ transplant recipients: A review of current evidence. Vaccine.

[57] Sabharwal C., Sundaraiyer V., Peng Y., Moyer L., Belanger T.J., Gessner B.D., Jodar L., Jansen K.U., Gruber W.C., Scott D.A. (2022). Immunogenicity of a 20-valent pneumococcal conjugate vaccine in adults 18 to 64 years old with medical conditions and other factors that increase risk of pneumococcal disease. Hum. Vaccines Immunother..

[58] Kobayashi M., Leidner A.J., Gierke R., Farrar J.L., Morgan R.L., Campos-Outcalt D., Schechter R., Poehling K.A., Long S.S., Loehr J. (2024). Use of 21-Valent Pneumococcal Conjugate Vaccine Among U.S. Adults: Recommendations of the Advisory Committee on Immunization Practices—United States, 2024. MMWR Morb. Mortal. Wkly. Rep..

[59] Froneman C., Kelleher P., José R.J. (2021). Pneumococcal Vaccination in Immunocompromised Hosts: An Update. Vaccines.

[60] van Heuvel L., Caini S., Dückers M.L.A., Paget J. (2022). Assessment of the inclusion of vaccination as an intervention to reduce antimicrobial resistance in AMR national action plans: A global review. Glob. Health.

[61] Yemeke T., Chen H.-H., Ozawa S. (2023). Economic and cost-effectiveness aspects of vaccines in combating antibiotic resistance. Hum. Vaccines Immunother..

[62] Ozawa S., Chen H.-H., Rao G.G., Eguale T., Stringer A. (2021). Value of pneumococcal vaccination in controlling the development of antimicrobial resistance (AMR): Case study using DREAMR in Ethiopia. Vaccine.

[63] Candeias C., Almeida S.T., Paulo A.C., Simões A.S., Ferreira B., Cruz A.R., Queirós M., Touret T., Brito-Avô A., de Lencastre H. (2024). Streptococcus pneumoniae carriage, serotypes, genotypes, and antimicrobial resistance trends among children in Portugal, after introduction of PCV13 in National Immunization Program: A cross-sectional study. Vaccine.

[64] Peela S.C.M., Sistla S., Nagaraj G., Govindan V., Kadahalli R.K. (2024). Pre- & post-vaccine trends in pneumococcal serotypes & antimicrobial resistance patterns. Indian J. Med. Res..

[65] Løchen A., Croucher N.J., Anderson R.M. (2020). Divergent serotype replacement trends and increasing diversity in pneumococcal disease in high income settings reduce the benefit of expanding vaccine valency. Sci. Rep..

[66] Balsells E., Dagan R., Yildirim I., Gounder P.P., Steens A., Muñoz-Almagro C., Mameli C., Kandasamy R., Lavi N.G., Daprai L. (2018). The relative invasive disease potential of Streptococcus pneumoniae among children after PCV introduction: A systematic review and meta-analysis. J. Infect..

[67] Grant L.R., Slack M.P.E., Theilacker C., Vojicic J., Dion S., Reinert R.-R., Jodar L., Gessner B.D. (2023). Distribution of serotypes causing invasive pneumococcal disease in children from high-income countries and the impact of pediatric pneumococcal vaccination. Clin. Infect. Dis..

[68] Grant L.R., Hanquet G., Sepúlveda-Pachón I.T., Theilacker C., Baay M., Slack M.P., Jodar L., Gessner B.D. (2024). Effects of PCV10 and PCV13 on pneumococcal serotype 6C disease, carriage, and antimicrobial resistance. Vaccine.

[69] Brooks W.A., Chang L.-J., Sheng X., Hopfer R. (2015). Safety and immunogenicity of a trivalent recombinant PcpA, PhtD, and PlyD1 pneumococcal protein vaccine in adults, toddlers, and infants: A phase I randomized controlled study. Vaccine.

[70] Salod Z., Mahomed O. (2022). Mapping Potential Vaccine Candidates Predicted by VaxiJen for Different Viral Pathogens between 2017-2021-A Scoping Review. Vaccines.

[71] Parveen S., Subramanian K. (2022). Emerging roles of extracellular vesicles in pneumococcal infections: Immunomodulators to potential novel vaccine candidates. Front. Cell. Infect. Microbiol..

